# Outcomes of a funding initiative to promote allied health research activity: a qualitative realist evaluation

**DOI:** 10.1186/s12961-020-00572-2

**Published:** 2020-06-19

**Authors:** Joanne Hilder, Sharon Mickan, Christy Noble, Kelly A. Weir, Rachel Wenke

**Affiliations:** 1 Allied Health Clinical Governance, Education and Research, Gold Coast Hospital and Health Service, Southport, QLD Australia; 2 School of Allied Health Sciences, Griffith University, Gold Coast, QLD Australia; 3 Faculty of Health Sciences and Medicine, Bond University, Gold Coast, QLD Australia; 4 School of Pharmacy and Office of Medical Education, The University of Queensland, Brisbane, QLD Australia; 5 School of Medicine, Griffith University, Gold Coast, QLD Australia; 6 Office of Medical Education, Faculty of Medicine, Herston Road, Herston, QLD 4005 Australia; 7 Menzies Health Institute, Griffith University, Gold Coast, QLD Australia

**Keywords:** Allied health, research, protected time, research capacity

## Abstract

**Background:**

Providing funding for clinicians to have protected time to undertake research can address a commonly cited barrier to research – lack of time. However, limited research has evaluated the impact or mechanisms of such funding initiatives. In the current economic environment, it is important that funding is used efficiently and judiciously and that mechanisms and contexts that may assist with maximising outcomes of funding initiatives are identified. This study aimed to describe the medium-term outcomes of a funding initiative to promote allied health research activity and to identify the key mechanisms and contexts that facilitated these outcomes.

**Methods:**

We used a qualitative research design informed by a realist evaluation, to conduct 10 semi-structured interviews with allied health professionals who had participated in a funding initiative 1–3 years ago. Questions explored outcomes, mechanisms and contexts of the funding initiative. Data was thematically coded into context–mechanism–outcome configurations.

**Results:**

Medium term outcomes included increased individual research opportunities, influence on team research culture and impact on clinical work/practice. Other outcomes included increased clinician confidence, knowledge and skill, and research outputs. However, some participants still had difficulties progressing research. Four context–mechanism–outcome configurations were identified to explain which contexts and mechanisms produced these outcomes. Examples of contexts included perception of managerial support, undertaking a research-based higher degree and joint applications, while mechanisms included accessing infrastructure and resources as well as individual researcher factors like motivation.

**Conclusion:**

Providing funding to allied health professionals to undertake and complete research can lead to important outcomes, including increased research opportunities, capacity and culture, increased research outputs, and changes to clinical practice. Outcomes are influenced by unique contexts and mechanisms and these should be considered in future implementation of similar funding initiatives.

## Introduction

Allied health professionals (AHPs) working in healthcare settings are well placed to produce and implement research evidence and building the research capacity of AHPs has been recognised as an international priority [1, 2]. A systematic review of allied health research culture found that, whilst AHPs are interested in undertaking research, they experience barriers, including having limited research skills and time [3]. For example, Pager et al. [4] found that AHPs are motivated to do research in order to develop skills, increase job satisfaction and address identified problems, while barriers to research engagement included lack of time, skills and having other work priorities. Emergent strategies addressing some of these barriers include increasing research skills and capability through specific education initiatives [5], collaboration with universities and employing research facilitators within the healthcare service to provide guidance and support [6–9].

Protected time away from clinical duties is another strategy that can address the often-cited barrier to research of reduced time. Ahmed et al. [9] reported, in their systematic review of research capacity-building interventions for health professional clinical educators (i.e. physicians, nurses, dentists, veterinarians and other AHPs), that protected time was a key strategy to improve research capacity. Protected time for research activities is an important strategy currently used in the United Kingdom to embed research into the core business of health services [10] and has also been recommended as a practical strategy in Australia [11].

Previous studies have reported small amounts of funding or bursaries that have allowed health professionals to have protected time for research and may be a cost-effective investment for building research capacity [12, 13]. For example, Lee and Saunders [13] provided small bursaries to clinicians leading 19 primary healthcare research projects, including three AHPs, and found that the bursaries produced research outputs, including six published papers. Other outcomes reported included skill and career development, where most recipients believed their research experience would contribute to further career opportunities [13]. Ried et al. [12] also provided 24 bursaries, 11 writing grants and 3 research fellow positions to primary care health clinicians. Grant holders were mostly AHPs (47%), although other health, medical and non-medical professions were also included. Two-thirds of the applicants reported that, following the funding, they had disseminated their findings either through publication or conference presentations. All grant recipients indicated that their funding scheme had positively impacted their interest to pursue further research [12]. While recent studies are reporting positive findings following protected time for research, they report a mixture of allied health and other professions (e.g. medicine) and therefore the outcomes of allied health-specific funding initiatives for protected time remain unclear.

Few studies have specifically evaluated impact of grant-funding initiatives to support offline time for allied health clinicians on research capacity-building. Hulcombe et al. [1] from Queensland, Australia, described the process of research grant funds, with a combined value between AUD$ 300,000 and AUD$ 700,000 per year amongst recipients, available to AHPs state-wide. Results showed an increase in the number of high-quality applications and novice applications received between 2009–2010 and 2013–2014 [1]. While an evaluation of the funded research positions indicated positive benefits [14], to date, no formal evaluation of the impact of these research grants has been reported.

More recently, Wenke et al. [15] implemented a funding initiative in an Australian tertiary regional health organisation, providing up to 20 days backfill funding to allow AHPs protected time to conduct research during their usual clinical hours. The initiative, which ran biannually, was established in 2014 and has provided funds to 41 AHPs across 7 rounds of funding to date. Wenke et al.’s [15] initial evaluation of the funding initiative for 16 AHPs found that the initiative significantly increased AHP’s self-reported research capacity on the Research Capacity and Culture tool [16]. It also led to a number of research outputs, including 11 articles submitted for publication, 4 successful ethics submissions and 3 studies commencing data collection [15]. Wenke et al. [15] also reported that, in the short-term, this funding initiative increased clinician’s research capacity and outputs. However, it is not clear how the local context, resources and infrastructure supported or hindered the achievement of outcomes. It also remains unclear what the medium- and longer-term outcomes of the initiative are. In Canada, a multi-level strategy to improve research capacity in a health service reported key outcomes that were categorized as either short-term (1–2 years), medium-term (3–5 years) or long-term (>5 years) outcomes [17]. This strategy did not specifically include protected time for research but one component did incorporate internal grants. They reported medium outcomes across the organisation, including increased research quality and increased research dissemination. Individual outcomes of researchers were not reported.

While investment in providing clinicians with protected time to undertake research has shown short-term improvements in research capacity and research outputs [12, 13, 15], research into the medium-term outcomes of such funding initiatives or the mechanisms that mediate such outcomes has not been reported. As governments and health services invest substantial funds into research grants to enable clinicians to undertake research, it is important that mechanisms likely to assist with maximising outcomes of the funding initiative are identified. Studies are also yet to investigate the barriers and enablers of using short-term funding initiatives to produce research outcomes in a healthcare context.

### Study objectives

This research aimed to evaluate the medium-term outcomes (i.e. 12 months to 3 years) of a supported allied health research funding initiative, including its impact on clinician’s research activity, capability and culture as well as what mechanisms helped or hindered achieving these outcomes. The specific research questions were:

1. What are the medium-term outcomes of a supported funding initiative to promote allied health research activity in relation to research capacity, outputs and engagement?
2. What are the mechanisms and context that can facilitate or hinder the outcomes of the funding initiative?

## Methods

We conducted a qualitative study informed by a realist evaluation. Semi-structured qualitative interviews were conducted to evaluate how the funding initiative worked, for whom and in what circumstances. Interviews were held from a minimum of 12 months to 3 years after the participants were awarded the funding for research.

### Participants

Thirty-four AHPs were awarded the funding between August 2014 and February 2017. Using purposive sampling, 13 AHPs who participated in the initiative were invited to participate in the study and 10 agreed to participate. An independent researcher, who was not involved in the funding initiative, emailed the eligible participants a participant information consent form and invited them to participate in the study. Ten AHPs agreed to participate in the study. Reasons for which participants declined to participate were high workload commitments, no longer engaged in the research project or feeling unable to provide additional insights about the initiative. A sample of 13 participants were purposively recruited to ensure the representation of participants across funding rounds, from different professions and with varying lengths of clinician experience. We also purposively included participants who were allocated the funding but did not use the funds to ensure a diverse range of experiences were captured [18].

### Funding initiative

The allied health funding initiative was open to all AHPs within the health service (approximately 880) and provided up to 4 weeks (full-time equivalent) of funding to enable the AHP’s clinical role to be undertaken by another clinician so that they could dedicate their time to a research activity. Access to funding and leave was flexible and depended on what was operationally suitable for continuity of their clinical workload. Applicants could apply to take the 4 weeks leave in one block, in multiple shorter blocks or part time, for example, 1 day a week for 20 weeks. This choice was often negotiated with their line manager to accommodate an operationally convenient backfill. An initial expression of interest involving a two-page application form was sent out via email to all AHP managers to disseminate to their teams. Research activities eligible for consideration included writing an ethics application, analysing and/or collecting data, undertaking a systematic review, and writing up research findings for publication. All applicants required endorsement from their line manager before applying. Applications were independently scored by two to three reviewers, with an allied health research and/or workforce background, according to predetermined criteria. Successful applicants were then paired with an Allied Health (AH) Research Fellow based at the health service. Initially, the AH Research Fellow met with the clinician to devise an implementation plan. During the funded time, clinicians were contacted by the AH Research Fellow to offer support and troubleshoot current or potential barriers. Where clinicians were undertaking research projects within existing research teams, mentorship was also received from these existing collaborations. The approximate cost per application ranged from AUD $5866–9666, with an average of AUD $8080.

### Data collection

Participants took part in a single face-to-face, in-depth interview with JH, an independent AHP who had been working as a research assistant for 12 months prior to the study. Interview questions were developed based on Cooke’s framework for research capacity-building (2) and explored the themes outlined in Wenke et al. [15]. Interview questions were emailed to participants at least 1 week before the interview to allow time for reflection. Reflective field notes were taken by the interviewer (JH) to enhance reflexivity [19]. Questions explored the medium outcomes of the funding initiative as well as what mechanisms and contexts helped or hindered the outcome as per the Realist Evaluation methodology [20]. Interviews were audio recorded, de-identified and transcribed by a professional transcription service. Interviews took approximately 30–45 min, with a median length of 40 min, and were undertaken at a convenient time for the interviewee within their work area. The interview guide is provided in Supplementary file 2.

### Data analysis

The first author JH used NVivo [21] software to code data from all transcripts. A deductive coding framework based on Realist Evaluation [20] methodology was used and focused on context–mechanism–outcome (CMO) configurations. Outcomes were self-reported and defined by the participants. Mechanisms were understood as factors that helped or hindered the outcome. Contexts were identified during team discussions and perceived as the circumstances that enabled the mechanisms. Findings were discussed with RW and then with the wider research team. Similar codes were merged and main themes and sub themes were identified using an iterative decision-making process. Discrepancies between the researchers were discussed until a consensus was reached.

## Results

### Participants

We conducted 10 interviews in April and May 2018 with AHPs who had participated in the funding initiative between August 2014 and February 2017. Participants were predominately female (*n* = 9) and represented 6 allied health professions, including physiotherapy, social work, occupational therapy, speech pathology, psychology and dietetics (Table 1). Participants worked across both inpatient and community practice settings. Half (*n* = 5) were base grade clinicians and the remainder were either senior or team leader clinicians. All clinicians were novice researchers with no or minimal experience in research. Most participants completed ethics applications (*n* = 4) or manuscripts for publication (*n* = 4) while they accessed the funding initiative. During the funding initiative time, participants undertook tasks such as writing an ethics application, writing up a manuscript or undertaking a systematic review (Table 2).

**Table 1:** Participant details

Participant details (*n* = 10)	Participants (*n*)
Male	1
Female	9
Funding round	
Round one (Aug-14)	2
Round two (Feb-15)	1
Round three (Aug-15)	2
Round four (Jan-16)	2
Round five (Aug-16)	2
Round six (Feb-17)	1
Profession	
Physiotherapy	3
Social work	1
Occupational therapist	1
Speech pathology	2
Psychology	1
Dietitian	2
Work setting	
Inpatient	8
Community	2
Clinical experience	
Base grade clinicians (entry level)	5
Senior health clinicians	4
Manager clinicians	1
Research-based higher degree student	3

**Table 2:** Tasks undertaken by participants

Primary task undertaken	Planned	Completed
Ethics application	4	2
Write-up of manuscript	4	4
Systematic review	2	2

Medium-term outcomes included research outputs, influence on team research culture, increased confidence, knowledge and skill, increased individual research opportunities, impact on clinical practice and difficulties progressing research. (These medium-term outcomes occurred after the funding initiative had been implemented and are described below followed by their CMO configurations. Sub-themes for these outcomes can be found as a supplementary file 3.).

#### Research outputs

All four of the participants who used the initiative to write a journal article were able to complete this task during or immediately after the initiative. To date, two of the journal articles have been accepted and published.

“*I guess that probably the biggest outcome was at the end of the four weeks we had a written paper that was able to be sent.*” (P5)

Three of the participants have presented their research at national, international conferences or local research forums.

While four participants used the initiative to work on ethics applications, only two participants submitted an ethics application during or immediately after the initiative. Of the submitted ethics applications, both studies are now collecting data.

Participants who were undertaking a systematic review completed part of the tasks that they had aimed to during their funded time. They continue to progress their review.

#### Influence on team research culture

Participants reported following the funding initiative that they were positively influencing and supporting research within their clinical teams. All participants said that they had already or would encourage other AHPs to apply for the research funding initiative because it helped to progress research projects. Participants were also supporting and encouraging other team members to do research by acting as knowledge brokers and sharing research networks.

“*… after that* [funding initiative] *I did feel confident to say if there was a new research project, or someone was talking about research, I would be able to guide them or talk about … the steps that you have to do.*” (P2)

One participant explained that she was now a role model within her team for new graduates who were interested in undertaking research and hoped she would encourage more new graduates to do so.

“*… the team could see that although I wasn't a really experienced* [professional]*, that I could still participate in the research process, it shows that anyone can be a part of research … there's not a barrier to novice clinicians wanting to help out … if I can do it, you know, anyone can. You don't have to be so many years out before you can put your hand up to be a part of a research group.*” (P6)

#### Increased confidence, knowledge and skill

Seven participants reported that they had an increase in their confidence, knowledge and skill to undertake or lead research after participating in the initiative.

“*… I definitely have more confidence in being able to do research projects on my own or just start getting the ball rolling*.” (P8)

The most common mechanisms facilitating participants’ research capabilities were having research fellow support, library support and, where relevant, undertaking a systematic review workshop.

Three participants described how their critical appraisal skills had also improved after undertaking the initiative.

“*I now subconsciously critically appraise articles when I read them, which … was something I definitely didn't do prior to the review or being part of the review*.” (P6)

#### Increased individual research opportunities

After participating in the initiative, participants reported having increased research opportunities. Participants described how their research networks had increased to include the health service library, local universities and the research fellows. Participants provided examples of how they had used and shared these networks to progress research. In one example, a participant explained that the networks they had developed while undertaking the initiative had led to further opportunities and a university had contacted the AHP to enquire if the health service could be a site for a separate study they were running (P1). Over half of the participants (*n* = 6) stated that they had plans to undertake research activities in the future and one participant stated that undertaking the research initiative had supported their career progression.

“*The other thing - this isn't necessarily a research output, but what it helped me with is my career progression…in terms of that I'm now currently in a research officer role*.” (P2)

#### Impact on clinical practice

Three participants reported that the results of their funded research activity had led to a positive impact in clinical practice. For example, from completing a systematic review, clinicians were now providing accurate information to patients and families about the impact of a treatment offered (P6). Another study provided the validity of a treatment method and the cost effectiveness, which led to the programme being sustained in the clinical service.

“*It also gave us the ability to continue the programme, so the programme still runs today, we've been able to show that it was evidence-based and with 4 weeks of intervention… patients were able to sustain results to 12 months. It was shown that it could be more financially viable for the service as well, which was a big thing*.” (P5)

The specific research projects led by the participants interviewed were at different stages in the research process. As some of the studies were still being completed, these AHPs were hopeful that, once completed, their findings would result in changed practice within the health service.

#### Difficulties progressing research

Three participants experienced difficulties progressing their research. One participant had stopped progressing their research study as a suitably qualified clinician to provide leave cover could not be found; they stopped their research study soon after. The other two participants experienced challenges finding the time to work on the study once the 4-week full time equivalent backfill period had been completed; they have since sought alternative funding sources to continue their study.

#### Temporary increase in co-worker’s workload

Participants reported that, while they were on research leave, their co-workers experienced a temporary increase in workload, because their role was covered by staff with less clinical experience.

“*I think in the end he had to get a new grad locum to start …So that probably placed a bit of a higher burden on the rest of the team…*” (P2)

### CMO configurations

**Fig. 1:**
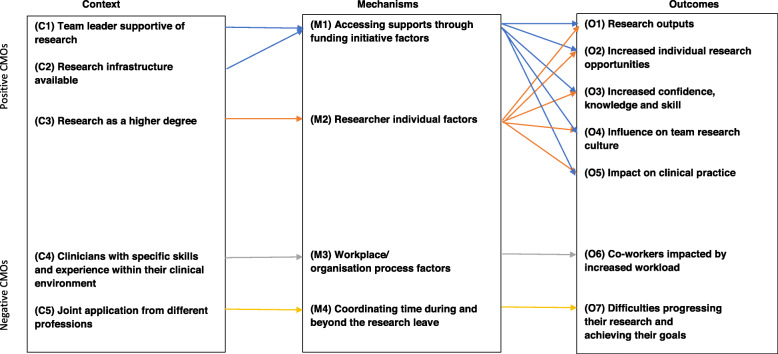
CMO configurations

Data analysis of the interviews produced four unique CMO configurations. There were a total of five contexts, four mechanisms and seven outcomes (Fig. 1).

Each CMO is explained in detail.

**CMO 1. AHPs who perceive their team leader to be supportive of research (C1), have research infrastructure available (C2) and are given time to access support (M1) produce research outputs (O1), have increased individual research opportunities (O2) and capabilities (O3), a positive influence on their team’s research culture (O4) and, in some cases, can change clinical practice (O5).**

The level of perceived support for research from the team leader was mentioned by all participants. The team leader support allowed AHP’s to use clinical time to engage with a wide range of infrastructure available to them, including research support and opportunities.

“*Our team leader was incredibly supportive of the process, so s/he was very keen and allowing* [of] *… time to put into the research or to go to meetings at the uni* [university] *or things which obviously helped greatly.*” (P5)

Support from the research fellow was accessed and highlighted by the AHPs as a facilitating mechanism. The participants reported that research fellows mentored them through their research, often providing examples of research materials from previous studies to reference or explain the research process.

“*Every time we’d have a meeting with* [the research fellow]*, she’d break it down into, these are the next steps, this is what we’re going to work on and so* [it] *just made it more achievable.*” (P10)

The hospital library was another support that was accessed by most participants. Participants used the library as a work-space to engage in research activities away from their clinical area to avoid interruptions as well as to access research software (EndNote and SPSS) and support in accessing the literature.

“*The librarians were really, really helpful particularly in terms of doing our literature search for us.*” (P7)

When participants were supported to access the available research infrastructure, participants could produce research outputs and increase their research opportunities and capabilities whilst also supporting their team’s research culture. Two of these participants used their research to change the clinical practice that they provided to patients (P5 and P6).

**CMO2. AHPs who undertake the funding initiative as part of a higher degree research (HDR) programme (C3) are motivated (M2) produce research outputs (O1), have increased individual research opportunities (O2) and capabilities (O3), a positive influence on their team’s research culture (O4) and, in some cases, can change clinical practice (O5).**

Three AHPs were undertaking research at the health service as part of a HDR programme. These participants were highly motivated to progress their research and at the same time progress their research degree.

“*I was quite motivated to get it done* [by] *having timelines of when it needed to be submitted, deadlines with the uni.*” (P8)

While some other participants experienced challenges in finding the time to progress their research past the funding initiative, HDR participants were driven by their degree and outcomes were not impacted by whether they perceived their team leader as supportive or unsupportive of research.

“*If I wasn't doing it as a PhD… to be completely honest, it barely would have moved from beyond clinical backfill time.*” (P9)

Participants who were HDR students also reported that they had increased their individual research networks and were continuing to undertake research. They reported having a positive influence on their team’s research culture by encouraging and supporting research within their clinical teams.

**CMO 3. Clinicians with specific skills and experience within their clinical environment (C4) who were backfilled by less experienced staff (M3) perceived their co-workers as being negatively impacted by the increased workload (O6) while they were doing their research.**

In some cases, it was challenging for suitable backfill to be found at the right time to match the specialist skills of some clinicians because there is often a limited number of employees who have the knowledge and skills to complete the role.

“*I was working in ICU* [intensive care unit] *at the time so it was hard to get someone with those skills to cover me to take me offline.*” (P8)

One solution that team leaders employed was to allocate a less experienced staff or new graduate to cover the clinicians’ clinical work. This resulted in other clinical team members having a temporary burden of increased workload during this time. It also meant, in some cases, that not all the clinician’s tasks were completed while they were backfilled and that they had to catch up when they returned to their clinical position.

“*Then when I came back … for 8 or 9 weeks* [I realised that] *my job hadn't been done at its full capacity, even though it could have been.*” (P9)

“*I did think there might have been a week or two delay, … in getting someone to backfill my position. ...I think in the end* [the team leader] *had to get a new grad locum to start, so it probably wasn't ideal from their perspective in that s/he had to do orientation and support their learning … when it was only for a* [short-term] *locum. That probably placed a bit of a higher burden on the rest of the team.*” (P2)

**CMO 4. Clinicians who prepared a joint application from different professions (C5) experienced difficulties coordinating their time during and beyond the research leave (M4) and progressing their research and achieving their goals (O7).**

In some cases, two AHPs working on the same research project applied to share the research initiative at the same time, each taking a portion of the 20 days leave available. These joint applications often involved AHPs from different professional teams. Research activities could only be completed when it was operationally viable for the clinical team. This resulted in clinicians having difficulties finding a mutually convenient time where they could be released from their clinical duties to work together on the research activity.

“*In terms of barriers to completing it, it is also because we are at that stage where* [the other AHP] *and I need to be together and you can only do that within work time really.*” (P7)

Additionally, some clinicians were required to rotate to another clinical area that had a different clinical population and was located in a different area, making it difficult to continue communicating. In these cases, research projects were either ceased or no progress was made for extended periods of time.

“*I think part of it also was because I’d moved from the area I was in, our research project initially was based around working in the ….* [unit]*, and then I had moved out of that area and was working in a different unit … because we weren’t practically based in the same area, whereas had we been, maybe there would’ve been some other ad hoc opportunities to do some of that.*” (P3)

## Discussion

The present study aimed to evaluate the medium-term outcomes of a supported funding initiative to promote research activity in AHPs and identify what mechanisms facilitated or hindered achieving these outcomes. We found seven medium-term outcomes of the funding, with the majority being positive, including increased individual research opportunities, positive influence on team research culture, increased confidence, knowledge and skill, increased research outputs, and impact on clinical practice. Another outcome of the funding, which reflected the challenge of undertaking research as a clinician, was that, at times, it was difficult to maintain research progress. Progress made during the backfill period was then stalled when the offline time was completed. Several contexts and mechanisms were found to influence these outcomes, which offer insight into the implementation of funding initiatives, such as this, in the future.

Previous research has reported that positive outcomes of bursaries and small grants for health professionals [12, 13, 15] include increased research outputs and capacity. Our team have also previously reported that a short-term clinical backfill funding initiative improved AHP’s individual confidence, knowledge and skills in undertaking research activities [15]. The present research is the first to date that has reported medium-term outcomes related to increased individual research opportunities, influence on team research culture and impact on clinical practice because of a funding initiative. These findings are important as, ultimately, health services want to improve outcomes for patients and a productive research culture within the health organisation may help to achieve this. Health services with a high research culture have been found to have benefits to patients (i.e. lower mortality rates), staff (e.g. reduced staff turnover), and productivity and efficiency [22]. Individuals who have increased opportunities and teams who have a positive research culture may be more likely to undertake research.

Some participants found it challenging to progress their research after the funding initiative had ceased. There is a risk that AHPs will only undertake research if they are able to secure similar grants or bursaries so they can undertake the research away from their clinical role. However, most participants were able to sustain their research after the funding initiative finished. To do this, many clinicians accessed ongoing support from Research Fellows. Ultimately, the funding initiative aims to develop a culture in which research activities, which are important to address clinical practice concerns, are partially funded, so that the research outcomes can be embedded into clinical practice. Current literature suggests that AHPs are motivated to undertake research but lack the time and skills to do so [4]. This funding initiative specifically addressed the time and support for AHPs to undertake research and one of the key outcomes was that participants increased their knowledge, skills and confidence in research while doing so. This study helps to explain how and why participants who undertook the funding initiative have increased their research capacity, therefore overcoming one of the barriers to research. This is consistent with United Kingdom research, where protected time (either by job planning or releasing staff from clinical duties) for research was an important strategy for embedding research activities into core business [10]. Health organisations should consider ways that they can provide time for clinicians to complete research projects as part of their core business.

Several of the mechanisms found to influence the outcomes of the initiative are also supported in the literature. For example, we found that AHPs needed the support of their team leader to be able to access the research infrastructure that is available to them at the health service and universities. This is consistent with other research, which has also identified that limited support from the workplace can be a barrier for clinicians undertaking research [12, 22, 23].

Similarly, Pager et al. [4] reported that AHPs were motivated to do research when it forms part of their postgraduate study. We found that AHPs who are undertaking a higher degree by research had increased motivation to progress and complete their research projects, which enabled them to overcome potential hindering mechanisms. We cannot know if participants who were HDR students would have achieved the same outcomes if they did not participate in the funding initiative. Research has shown, however, that HDR students who are studying part time or who do not have a scholarship are less likely to complete their studies [24]. Given that the HDR completion rate in Australia’s top universities is between 62% and 72% [25], we cannot assume that AHPs will complete their research without other supporting mechanisms. Other options that may increase motivation could be incorporating research engagement into the AHP’s professional development plan.

Participants who were undertaking the initiative in a joint application with another AHP found it difficult to coordinate time to work on the research, both during and beyond the research leave, which resulted in difficulties in progressing the research. Developing the skills needed to undertake research takes time and is dependent on the individual. This study supports recommendations made by Ried et al. [12], in that funding initiatives need to be flexible to support individual skill and project specificity. Therefore, when two or more AHPs work together on research they will likely require more time. In addition to this, managerial support and planning is required to ensure that both AHPs can access leave at the same time, particularly if they are working across different professions.

Short-term outcomes from the funding for research initiative have been published elsewhere [15], including increased individual research capacity using the Research Capacity and Culture Tool survey, high clinician satisfaction and increased research outputs. An additional short-term outcome found in this study was that co-workers of AHPs who undertook the funding initiative experienced a temporary increase in workload while the AHP was on research leave. Most teams found it difficult to find leave cover. In cases where a less-experienced staff was employed to cover the research leave, co-workers in the team were impacted by increased workload. While this impact was for a limited time, it may deter other AHPs from applying for the initiative.

### Implications

Our realist evaluation describes the contexts in which a funding for research initiative works best. To facilitate research capacity, funding initiatives may be more effective in a context where the AHP’s team leader is supportive of research and the health service has research infrastructure available. Additionally, consideration and time to plan for appropriate leave cover is required to limit the impact on co-workers. When AHPs access the support that is available, it leads to rich outcomes for the individual and the health service. These same outcomes can be achieved when AHPs undertake research through a higher degree by research programme. Therefore, when AHPs are planning to undertake a substantial research project, they should be encouraged to consider doing it as part of a higher degree or have an additional funding source identified, to enable completion. Additionally, AHPs who are working full time and undertaking a HDR programme would benefit from this initiative. When AHPs from different disciplines work together on a project, a plan needs to be made between the clinicians and the managers of the disciplines on when and how time can be coordinated so that the AHPs can work together on the research project. This will ensure that the research can continue, regardless of whether the research team needs to physically work together or not. To allow for time for managers to find an appropriately skilled clinician to undertake clinical duties during the research period, a longer amount of time to use the funding and early identification of projects may allow more time for leave cover recruitment. Future research could evaluate longer-term outcomes (5–10 years) to see if AHPs who undertook the funding initiative were successful in completing their research degrees, translating their findings into practice or obtain an academic position.

### Strengths and weaknesses of this study

While this study had a small sample size of 10, it included participants from a cross section of professions and clinical experience. While this is a small sample size, we considered it sufficient to answer our research questions. When determining a sample size, Malterud et al. [26] suggests that researchers should consider the aim of the study, sample specificity, if a theory is being used, the quality of the dialogue and the analysis strategy. Given that the aim of this study is narrow, we used purposive sampling to ensure that we had a wide range of participants and we used realist evaluation theory to interpret and understand the results of the study. This study was completed at one health service only. While we have outlined what works, for whom and in what context, we cannot presume that these outcomes will transfer to other health services. We encourage other health services to investigate and compare our recommendations with their own contexts. Other health professions, including medicine and nursing, may also be interested in implementing and evaluating the initiative. An unreported strength of this study is that three participants have reported that their research has positively impacted on clinical practice; for example, one study validated a dietetic treatment method, which led to the programme being continued in clinical services. It will be important for future research to evaluate how the research findings from this and other supported schemes are translated into practice. We expect there will be long lead times as some participants are still completing these research projects. In addition, future research should evaluate the cost effectiveness of similar bursaries to support research in healthcare settings.

## Conclusion

Providing supported funding to AHPs to complete research can lead to important outcomes, including increased research opportunities, capacity and culture. It can also produce research outputs and change clinical practice. However, the outcomes are influenced by key contexts, including managers and team leaders of AHPs being supportive of clinician’s undertaking research activities and the health service having research infrastructure in place. Mechanisms such as clinician engagement, clinician motivation, coordination of time and finding suitable leave cover can further enhance outcomes dependent on context. Due to the potential meaningful medium-term outcomes on research capacity and culture, health services should consider funding initiatives that allow AHPs to undertake research leave and consider the aforementioned factors when planning its implementation.

## Supplementary information

**Additional file 1.** Outcome sub-themes of funding for the research initiative.

**Additional file 2.** Interview guide.

**Additional file 3.** Outcome sub-themes of funding for research initiative.

## Data Availability

The interview guide is available as a supplementary file. Further data is available on request from the authors.

## Abbreviations

AH: allied health
AHP: allied health professional/s
CMO: context–mechanism–outcome
HDR: Higher Degree Research
