# Supplementary material for: Outcomes of a funding initiative to promote allied health research activity: a qualitative realist evaluation
Source: Health Res Policy Syst. 2020 Jun 19;18:71. doi: 10.1186/s12961-020-00572-2 (PMC7305620; doi:10.1186/s12961-020-00572-2)
Supplement: Supplementary file 1 — Additional file 1. Outcome sub-themes of funding for the research initiative. [file 12961_2020_572_MOESM1_ESM.docx]

**Supplementary file 1. Reported outcomes of bursaries in the literature**

| **Short term** | **Medium term** | **Long term** |
| --- | --- | --- |
| Increased research capacity  Research outputs [1] | Dissemination of findings [2-4] | Expected increased career opportunities [3] |
| Research skill development [1] | Increased interest in pursuing research [4] |  |
|  | Increase in grant applications [5] |  |
|  | Increased research quality [2] |  |

1. Wenke R, Weir KA, Noble C, Mahoney J, Mickan S: **Not enough time for research? Use of supported funding to promote allied health research activity.** *Journal of multidisciplinary healthcare* 2018, **11:**269.

2. Trytten C, Wale M, Hayes M, Holmes B: **Lessons learned from a health authority research capacity-building initiative.** In *Healthcare management forum*. SAGE Publications Sage CA: Los Angeles, CA; 2019: 259-265.

3. Lee M, Saunders K: **Oak trees from acorns? An evaluation of local bursaries in primary care.** *Primary Health Care Research & Development* 2004, **5:**93-95.

4. Ried K, Farmer EA, Weston KM: **Bursaries, writing grants and fellowships: a strategy to develop research capacity in primary health care.** *BMC Family Practice* 2007, **8:**19.

5. Hulcombe J, Sturgess J, Souvlis T, Fitzgerald C: **An approach to building research capacity for health practitioners in a public health environment: an organisational perspective.** *Australian Health Review* 2014, **38:**252-258.
