# Supplementary material for: Outcomes of a funding initiative to promote allied health research activity: a qualitative realist evaluation
Source: Health Res Policy Syst. 2020 Jun 19;18:71. doi: 10.1186/s12961-020-00572-2 (PMC7305620; doi:10.1186/s12961-020-00572-2)
Supplement: Supplementary file 2 — Additional file 2. Interview guide. [file 12961_2020_572_MOESM2_ESM.doc]

# Interview Guide

1. Describe briefly your involvement or interest in research.
2. What do you remember most about your experience with the clinical backfill funding for research?
3. What were the outcomes of you participating in the clinical backfill funding initiative?

Prompts: research outputs? (i.e., journal publications, conference presentations, ethics applications, successful grant funding)

Confidence/knowledge/skills in research?

Impact on clinical work?

Impact on your team?

Networks?

Future planed research activities?

Any other outcomes?

For each listed outcome, the interviewee will then be asked what helped or hindered each outcome and

Are you able to make a comment on the timeframes for these outcomes? i.e., which outcomes occurred more immediately after the backfill and which have taken longer to emerge?

1. Please describe how your confidence, knowledge and skills enabled you to achieve these outcomes?
2. Did you achieve everything you set out to during the time? What do you think helped/ hindered this?

Prompts: your own personal development skill development? (what helped or hindered)

1. Where there any barriers to undertaking research associated with the funding? And if so can you describe these?

Prompts: capability, opportunity, motivations

(any other barriers?)

1. Was there anything that assisted undertaking research during the backfill time that made it easier? If so, can you describe these?

(any other facilitators?)

1. It is now **[number]** years since you completed the backfill, what is the current progress on the project that you received funding to work on during the backfill time (i.e., publication, collecting data?) (consider how earlier mechanisms discussed in Q 2,3 may have contributed to ongoing progress or lack of progress)
2. Have you or would you encouraged other co-workers to consider applying for the funding initiative? Why/why not?
3. Do you have any suggestions of how the backfill funding initiative could be improved or sustained in the future?
4. anything else that you would like to add about the backfill funding initiative that we haven’t spoken about yet?
