# Supplementary material for: Outcomes of a funding initiative to promote allied health research activity: a qualitative realist evaluation
Source: Health Res Policy Syst. 2020 Jun 19;18:71. doi: 10.1186/s12961-020-00572-2 (PMC7305620; doi:10.1186/s12961-020-00572-2)
Supplement: Supplementary file 3 — Additional file 3. Outcome sub-themes of funding for research initiative. [file 12961_2020_572_MOESM3_ESM.docx]

**Supplementary file 3. Outcomes of funding for research initiative**

| Main theme | Sub-theme |
| --- | --- |
| Influence on team research culture | - Supporting and encouraging co-workers to do research - Encouraging co-workers to apply for the funding initiative - Other co-workers doing research - Sharing networks with co-workers - Role modelling as a new graduate undertaking research |
| Increased individual research opportunities | - Increased networks - Plans for research career progression |
| Impact on clinical work or practice | - Better information for patients - Program that was focus of research evaluation, continues to run - Expanding clinical practice based on systematic literature review findings |
| Research outputs | - Literature review - Ethics application - Publication - Conference presentation |
| Increased confidence, knowledge and skill | - Searching the literature - Appraising the literature - Undertaking research activities |
| Difficulties progressing research | - Unable to find clinician to provide leave cover - Challenges finding the time to work on the study post backfill period |
| Temporary increase in co-worker’s workload | - Co-workers experienced a temporary increase in workload |
